# Prospects of Biogenic Xanthan and Gellan in Removal of Heavy Metals from Contaminated Waters

**DOI:** 10.3390/polym14235326

**Published:** 2022-12-06

**Authors:** Katarína Balíková, Bence Farkas, Peter Matúš, Martin Urík

**Affiliations:** Institute of Laboratory Research on Geomaterials, Faculty of Natural Sciences, Comenius University in Bratislava, Mlynská dolina, Ilkovičova 6, 84215 Bratislava, Slovakia

**Keywords:** biosorption, heavy metals, heteropolysaccharides, remediation, wastewater

## Abstract

Biosorption is considered an effective technique for the treatment of heavy-metal-bearing wastewaters. In recent years, various biogenic products, including native and functionalized biopolymers, have been successfully employed in technologies aiming for the environmentally sustainable immobilization and removal of heavy metals at contaminated sites, including two commercially available heteropolysaccharides—xanthan and gellan. As biodegradable and non-toxic fermentation products, xanthan and gellan have been successfully tested in various remediation techniques. Here, to highlight their prospects as green adsorbents for water decontamination, we have reviewed their biosynthesis machinery and chemical properties that are linked to their sorptive interactions, as well as their actual performance in the remediation of heavy metal contaminated waters. Their sorptive performance in native and modified forms is promising; thus, both xanthan and gellan are emerging as new green-based materials for the cost-effective and efficient remediation of heavy metal-contaminated waters.

## 1. Introduction

Heavy metals are considered metallic elements (in some cases they comprise metalloids, such as arsenic) whose compounds can induce toxicity at a low level of exposure, thus posing a critical threat to human health [1,2,3,4,5]. Although they are naturally occurring, human and wildlife exposure to heavy metals has increased due to excessive anthropogenic activities that contribute significantly to environmental contamination (e.g., industrial production, mining, and smelting) [6]. On the other hand, they serve various key physiological and biochemical functions in organisms, e.g., copper is an important catalytic cofactor in redox-active enzymes [7]. Still, chronic low-dose exposure is a major public health concern, since heavy metals cannot be broken down and (some) have a bioaccumulative nature; hence, they are persistent in the environment [8].

Since heavy-metal-associated acute and chronic toxicity at low concentrations may pose a critical threat to human health [9], various in situ and ex situ remediation techniques have been developed to limit their transfer to the food chain [10]. These usually employ methods that aim to contain, clean up, or restore the heavy-metal-contaminated sites (soil, sediments, or waters) via various physical, chemical, or biological principles, e.g., adsorption, bioaccumulation, (phyto)extraction, or stabilization [11,12]. Still, there is continuous pressure to deploy innovative and site-specific remediation technologies which could feasibly and efficiently remediate contaminated matrices [13,14,15]. This also includes sustainable green remediation techniques, such as immobilization of contaminants using natural or waste-derived green materials, which provide various environmental and economic benefits in comparison to conventional approaches [16,17].

Regarding the issue of finding new green-based materials for the remediation of contaminated waters, biopolymers have emerged as a promising alternative to synthetic polymers, primarily due to their biodegradability, non-toxicity, and economic sustainability [18]. This review focuses on two biopolymers that are considered for application in various new green technologies (e.g., environment-friendly construction and development [19])—heteropolysaccharides xanthan and gellan. These have been used for several decades in the food industry, and, thus, their production is well understood, although their biochemical machinery less so. Recently, there have been also successful attempts to reduce the xanthan and gellan production costs and improve yields by utilizing alternative carbon and nutrient sources, e.g., the orange peel’s hydrolysate [20] and corn steep liquor [21]. This highlights the economic feasibility of xanthan- and gellan-based materials for the immobilization of heavy metals, if optimized for specific site conditions and contaminant characteristics.

## 2. Biosynthesis of Xanthan and Gellan

**Figure 1:**
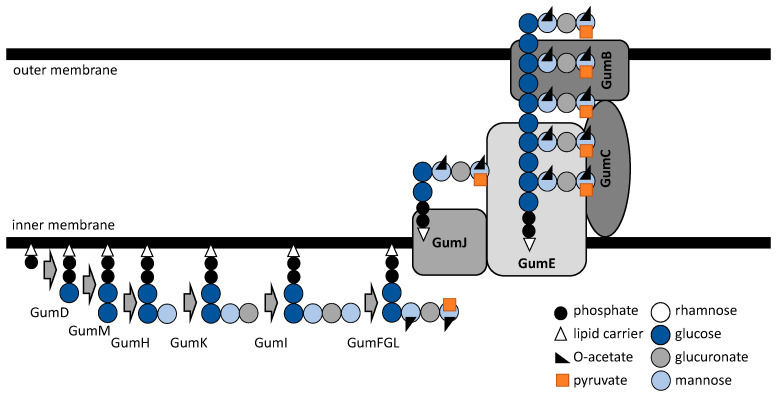
Schematic model of xanthan polysaccharide biosynthesis machinery.

**Figure 2:**
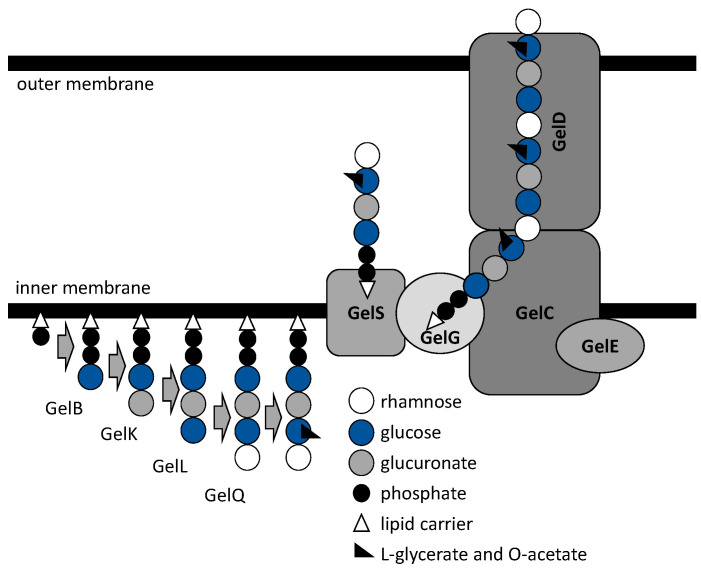
Schematic model of gellan polysaccharide biosynthesis machinery.

Xanthan and gellan biosynthesis comprises several steps (Figure 1 and Figure 2), including the synthesis of exopolysaccharide precursors, repeat-unit assembly on a lipid carrier located at the cytoplasmic membrane, its modification, membrane translocation, polymerization, and export [22]. Thus, there are several functionally distinguished enzymes required for exo(hetero)polysaccharide synthesis and development [23,24].

### 2.1. Xanthan Biosynthesis

Like most bacterial surface heteropolysaccharides, xanthan is synthesized through a Wzy-dependent pathway, where the lipid-linked repeat units are built up on polyisoprenol phosphate at the cytoplasmic face of the inner membrane, then flipped to the periplasmic face of the inner membrane by a flippase and polymerized by a block-transfer mechanism involving the Wzy polymerase [25].

The direct precursors for xanthan production in *Xanthomonas campestris* pv. *campestris* B100 are the nucleotide sugars GDP-mannose, UDP-glucose, and UDP-glucuronic acid, which comprise the xanthan repeating units (in a molar ratio of 2:2:1).

The building of pentasaccharide units is under the control of the 12 kb gene called the *gum* cluster. The *gum* cluster comprises 12 genes that are involved in the assembly of the pentasaccharide repeating unit, its decoration with substituents, polymerization, translocation, and secretion [26,27]. However, the key factors for the xanthan’s precursors’ biosynthesis are the genes *xanA* and *xanB*, which are not included in the *gum* cluster. The *xanA* and *xanB* genes encode bifunctional enzymes phosphoglucomutase/phosphomannomutase, which convert glucose-6-phosphate and mannose-6-phosphate to their corresponding 1-phosphates, and phosphomannose isomerase/GDP-mannose pyrophosphorylase, respectively [28]. Since *xanA* deletion restricted the capacity to synthesize the nucleotide, the monosaccharide composition of xanthan gum in *Xanthomonas citri* subsp. *citri* was affected. This resulted in the decrease of biofilm formation, sliding motility, and pathogenicity of this phytopathogenic Gram-negative proteobacterium [29].

The precursor biosynthesis is followed by the processes of building and decoration of pentasaccharide subunits at the cell envelope. Among others, the process is initiated by the GumD and GumM glycosyltransferases. While the former transfers glucose-phosphate residue from UDP-glucose to an undecaprenyl-phosphate C-55 lipid carrier molecule located at the cytoplasmic face of the membrane [30], the latter adds the second glucose residue to the molecule [31]. Mannose is then transferred to the assembling site by GumH (α-1,3-mannosyltransferase) from GDP-mannose [32], followed by a glucuronic acid residue addition by GumK (beta-1,2-glucuronosyltransferase) from UDP-glucuronic acid [33]. The pentasaccharide subunit is finalized by GumI (beta-mannosyltransferase) activity [34]. The outer, last-added mannose can be pyruvylated by GumL, and the GumG and GumF O-acetyltransferases allow the acetyling of both terminal and inner mannose residues of the repeating unit, respectively [26].

Finished lipid carrier-linked repeating pentasaccharide units are then translocated by the putative flippase GumJ to the periplasmic face of the inner membrane, where it can be polymerized by GumE, a homolog to O-antigen polymerase Wzy [23], which transfers immature xanthan polymers to newly translocated repeat units. Unfortunately, there is still little available evidence available regarding these two enzymes [35]. The transfer and excretion of xanthan across the bacterial membrane are regulated by *gumB* and *gumC* [36], whose overexpression yields xanthan molecules of higher molecular length. The lipoprotein GumB is associated with the outer membrane and is responsible for outer membrane polysaccharide export, while GumC is an inner membrane protein belonging to the polysaccharide co-polymerase family [37].

### 2.2. Gellan Biosynthesis

The activated sugar precursors for biosynthesis of tetrasaccharide unite in gellan, a sphingan heteropolysaccharide produced by genera *Sphingomonas*, include UDP-glucose, UDP-glucuronic acid, and deoxythymidine diphosphate (dTDP)-rhamnose [38]. The enzymes produced by *Sphingomonas* spp., which are presumed to be involved in precursor synthesis, include phosphoglucomutase catalyzing the conversion of glucose-6-phosphate into a glucose-1-phosphate encoded by *pgmG* gene and glucose-1-phosphate uridylyltransferase (pyrophosphorylase) encoded by the *ugpG* gene [22]. The latter, an UgpG protein encoded outside the *gel* cluster (18-gene cluster required for gellan biosynthesis), also exhibited dTDP-glucose pyrophosphorylase activity in *Sphingomonas elodea* ATCC 31461 [39]. The biosynthetic pathway of dTDP-L-rhamnose is highly conserved among both Gram-positive and Gram-negative bacteria [40].

The genes involved in the sequential assembly of repeating tetrasaccharide units of the gellan backbone include *gelB*, *gelK*, *gelL*, and *gelQ* encoding the glycosyl transferases [22]. The priming transferase, a glucosyl-isoprenylphosphatetransferase encoded by *gelB*, enables the transfer of UDP-glucose residue to an activated C_55_-isoprenylphosphate lipid carrier. The gene *gelB* is homologous to *spsB* from *Sphingomonas* sp. ATCC 31554 encoding glucosyl-isoprenylphosphate transferase [24]. The GelK is a *β*-1,4-glucuronosyltransferase, structurally homologous to *E. coli* N-acetyl glucosaminyl transferase MurG, which catalyzes the transfer of UDP-glucuronate residue to activated lipid link glucose [41]. The remaining glycosyl transferases are coded by *gelL* (homologous to *spsL*) and *gelQ* of the gellan cluster, which most likely enable the incorporation of the third (glucose) and fourth (rhamnose) sugars to the tetrasaccharide repeating unit of the gellan exopolysaccharide, respectively [42,43]. The repeating unit is finalized by the substitution at the first glucose unit catalyzed by enzymes having acetyltransferase and glyceryltransferase activities.

The genes *gelS* and *gelG* (homologues to *Wzx* and *Wzy*) located in the *gel* cluster region IV are likely involved in the repeating unit translocation across the inner membrane and polymerization of gellan, respectively. Its chain-length-regulating protein, a polysaccharide co-polymerase, is encoded by the genes *gelC* and *gelE*, with the latter reportedly having an ATP-binding activity in vitro [44]. Finally, the gellan chains’ secretion process is regulated by the integral outer membrane auxiliary homologue protein encoded by the gene *gelD* [45].

### 2.3. Prospects of Genetic Engineering in Heavy Metal Remediation Using Xanthan and Gellan

There are various emerging molecular techniques which address heavy metal pollution and which, via genetic engineering, exploit some of the metal-related features of bacterial metabolism, e.g., synthesis of the specific metal-chelating polymers [46]. Engineered enhancement of targeted gene expression in the xanthan and gellan biosynthesis pathways should improve the desired bacterial feature for heavy metal removal from contaminated waters (e.g., polysaccharide quality or yield), as well as the competitiveness of the “green” polysaccharide-based adsorbent with the other commercially available alternatives.

Identifying the components responsible for the pollutant binding as well as the understanding of the molecular machinery of their biosynthesis are both essential aspects for the successful implementation of the genetic engineering principles in enhancing the performance of xanthan and gellan in remediation of heavy metal-contaminated waters, as well as in reducing the costs for the polysaccharides’ production. Unfortunately, to the best of our knowledge, there is no molecularly oriented research on xanthan and gellan synthesis that directly relates to remediation of heavy metal contaminated sites. Still, the development of the low-cost, high-yield production of recombinant biopolysaccharides is highly beneficial for food, pharmaceutical, and cosmetic industries; thus, a lot of progress has been made regarding the genetic engineering in these industrial areas.

In the effort to enhance gellan production, Manjusha et al. [47] successfully cloned four gellan gum genes (*gelQ*, *gelK*, *gelL*, and *gelB*) and produced the recombinant polysaccharide that was similar in structural properties with the native gellan. Transformation of the recombinant pBBR122-*gelD* plasmid into *Sphingomonas paucimobilis* cells and overexpression of the *gelD* in recombinant strain yielded twice as much gellan in comparison to a wild-type strain [48]. Via exploitation of genetic engineering, it is also possible to synthesize unnatural polymers, or the structural variants of natural xanthan or gellan via constructing mutants defective or altered in the polysaccharide biosynthetic pathway [49]. Since polymers of mutated strains differ in the chemical and physical properties in comparison to products of wild-type strains (e.g., acetylation and pyruvylation patterns) [50], their altered performance in the remediation of heavy-metal-contaminated waters can be also expected. However, targeting only the genes directly involved in synthesis of polysaccharides may not be sufficient. Sá-Correia et al. [22] noted that individual increases in *pgmG*, *ugpG* or *gelK* gene copies in recombinant plasmids had no positive effect on gellan productivity, while the simultaneous overexpression of *pgmG* and *ugpG* genes that are involved in precursors synthesis increased the overall production of gellan by 20%. Another approach, which reduces the cost of xanthan and gellan production and is unrelated to genes directly involved in polysaccharide synthesis, includes constructing mutants that produce more polysaccharides and which are capable of utilizing a cheap industrial by-product, e.g., construction of transconjugants which utilize whey- or lactose-containing media for xanthan production [51].

It is reasonable to expect that the ever-growing versatility and precision in genetic engineering techniques will, in time, enable the modification of the xanthan and gellan biosynthesis machinery to an extent that the capacity of microorganisms to tolerate and accumulate heavy metals will be enhanced significantly, and they can be employed for the development of a green clean-up strategy that utilizes the recombinant polysaccharides for the removal of heavy metals from waters.

## 3. Xanthan- and Gellan-Producing Bacteria

Xanthan gum is an extracellular water-soluble heteropolysaccharide that is commercially produced from various carbon sources by fermentation in controlled conditions using a Gram-negative bacteria *Xanthomonas campestris* belonging to the family *Pseudomonadaceae* [52]. Being discovered in the 1950s, it belongs to one of the earliest marketed bacterial exopolysaccharides that have been accredited for food use in the USA [53]. Although *X. campestris* is most commonly employed for industrial production of xanthan gum, there are several other strains of genus *Xanthomonas* which can produce xanthan, e.g., *X. arbicola* pv. *juglandis*, *X. axonopodis* pv. *begonia*, *X. axonopodis* pv. *vesicatoria* [54], and *X. pelargonii* [55].

Gellan (earlier referred to as polysaccharide S-60), a sphingan group of heteropolysaccharides, is secreted by members of the bacterial genus *Sphingomonas*, which belongs to the α-4 subclass of the *Proteobacteria* [56]. It is manufactured commercially from the late 1980s by aerobic fermentation of *Sphingomonas paucimobilis* ATCC 31461 strain (originally designated *Pseudomonas elodea*; also referred to as *Sphingomonas elodea*), a highly productive industrial strain of gellan gum. There have been other strains of *S. paucimobilis* reported to be capable of producing gellan gum [57], including *S. paucimobilis* ATCC 31461 [58] and *S. azotifigens* GL-1 [35]. Various recent studies have focused on the use of abundant and inexpensive waste products for gellan production, e.g., Jin et al. [59] developed a low-cost fermentation medium using soybean pomace as a nitrogen source. It was successfully applied for fermenting a UV-induced mutant of *S. paucimobilis* ATCC 31461, strain NK2000. Raghunandan and Kumar [60] tested ten *Sphingomonas* bacteria isolated from biodiesel-derived waste glycerol for their potential usage in the commercial production of gellan. Two isolates, *S. yabuuchiae*, and *S. pseudosanguinis*, displayed high waste glycerol degradation as well as gellan conversion rates. They demonstrated great potential in the cost-effective commercial production of gellan, and great prospects in the remediation of biodiesel-derived crude glycerol.

### Potential of Xanthomonas and Sphingomonas Bacteria in Heavy Metal Removal

Exposure to heavy metals can induce various physiological responses in bacteria, such as alteration in gene expression related to the transport of metal cations and oxidative stress, or it can enhance the production of extracellular polymers capable of sequestering the toxicants. Banjerdkij et al. [61] noted that *X. campestris* is sensitive to cadmium’s toxic effects. However, pre-exposure to low concentrations of cadmium(II) induced cross-protection against the lethal concentrations of cadmium(II) and zinc(II). Similar effects have been reported by Hrimpeng et al. [62] in the case of pre-exposure of *X. campestris* to sublethal concentrations of arsenic(III), which induced the cross-protective response to CuOOH and peroxide. Cadmium(II) has been also shown to be a potent inducer of peroxide-scavenging enzymes [63]. These scavengers are pivotal for the survival of the phytopathogenic fungi, since plants induce oxidative stress in infecting bacteria by generating ROS, such as peroxide. Since the metal-induced oxidative stress in cells can be partially responsible for the toxic effects of heavy metals, the frequent use of metal-containing bactericides by farmers may affect the appearance of heavy-metal-resistant strains of *Xanthomonas* in populations of plant-pathogenic bacteria [64]. However, their induced resistance is highly advantageous in the case of ex situ remediation of heavy-metal-contaminated waters by bioaccumulation, a cost-effective and environmentally beneficial remediation technique, which uses the living cells under regulated conditions, e.g., in bioreactor systems [65].

Also, *Sphingomonas* sp. LK11 strain has shown promising prospects in the remediation of contaminated environments, since it maintained steady growth during cadmium-induced stress. However, Li et al. [66] reported that the main mechanism of cadmium(II) removal by *Sphingomonas* sp. was biosorption, and the involvement of carboxyl and carbonyl functional groups of saccharides in cadmium immobilization via physical entrapment, ion exchange, and complexation has been suggested. Hence, the metal binding most likely occurred on extracellular polysaccharides. Furthermore, Ashour [67] noted that cobalt(II) has been shown to enhance the exopolysaccharides’ production by *X. campestris* This physiological response of bacterium to heavy metal exposure can be viewed as a protective mechanism against cobalt’s toxic effects, since the sequestration of the metallic cations inhibits their entry into the cells [68].

The Involvement of xanthan and gellan in heavy metal cation sequestration by living bacteria is plausible, since both polymers exhibit anionicity, and they are packed with negatively charged functional groups [69]. For example, xanthan, surrounding the cells of *Xanthomonas* spp., is expected to play a substantial role in metal removal from the water produced by the oil industry via binding the metal cations by forming the Werner-type complexes, since it possesses a number of hydroxyl and carboxyl groups [70]. However, the polysaccharide synthesis pathway is sensitive to stress conditions, and, thus, depending on the concentration and speciation of the heavy metal, the performance of the bacterium in remediation of contaminated medium can be hindered. Although there are reports indicating that the low metal concentration may be beneficial for the metal sequestration [67] and that it may enhance the survival rate of the bacterium in contaminated environments [61], the immobilization capacity of the metabolically active bacteria in bioreactors during wastewater treatment has its limitations under high metal loading conditions due to imminent detrimental effects of toxic metallic cations on bacterial physiology, cell’s structural integrity, and essential metabolic processes. Still, the relatively high abundance of *Sphingomonas* genera in soil microbial communities at sites highly contaminated with heavy metals due to long-term smelting and mining operations [71] renders these Proteobacteria a suitable candidate for bioremediation. Similarly, since *Xanthomonas* bacteria possess effective enzymatic and non-enzymatic strategies to survive and proliferate in the presence of reactive oxygen species and, thus, have shown great tolerance to heavy-metal-dependent induction of oxidative stress in cells [63], their prospects for direct application in bioremediation technologies should be also positive. However, their actual performance in metal removal does not necessarily correlate well with the strain’s survival rate, since a mechanism other than metal sequestration can be involved, e.g., upregulation of copper-inducible *copAB* operon identified in some of *Xanthomonas* species that regulate efflux of copper [72].

Since bacterial sensitivity to heavy metals and their specific growing and nutritional requirements (e.g., optimum pH, presence of carbon and nitrogen sources) provide drawbacks to their feasibility, and xanthan and gellan have performed well in heavy metal immobilization, the utilization of separated bacterial polysaccharides in decontamination of wastewaters can be advantageous over living cells. This is most likely the reason why there are virtually no comprehensive studies on bioaccumulation of heavy metals by xanthan- or gellan-producing living bacteria. That being the case, the more complex kinetic and thermodynamic studies on heavy metal removal are available only for the dead cells, e.g., the report on copper(II) biosorption by non-living biomass of *S. paucimobilis* [73]. Still, the involvement of mechanisms other than physicochemical sorptive interaction may allow the living cells to outperform the pure extracellular polymers or dead biomass in metal separation, e.g., the ability of mercury-resistant bacteria belonging to *Xanthomonas* to reduce mercury(II), thus accelerating the mercury volatilization [74].

## 4. Chemical Properties of Xanthan and Gellan Associated with Their Sorptive Performance

Both the xanthan and gellan chains are considered stable in aqueous solutions and capable of reversibly binding the metallic ions. Their ability to bind cations is due to the presence of various active functional groups. FTIR spectra of xanthan gum revealed the presence of a prominent broad band typical for hydroxyl groups, but also carbonyl esters of acetyl and carboxylate groups [75]. Similarly, spectroscopic analysis of gellan usually reveals the presence of hydroxyl and carboxylic groups, as well as alkyl ether [76].

The xanthan molecule is composed of pentasaccharide repeating units which consist of a cellulose-like backbone with a trisaccharide side-chain of two mannose and one glucuronate residue attached to a β-D-glucose unit, with pyruvic acid linked to O(4) and O(6) of the terminal D-mannose [77] and an acetyl group positioned at O(6) of the main chain D-mannose unit. The abundance of pyruvate and acetal groups of xanthan depends on the medium composition, the *Xanthomonas* strain, as well as the operational conditions used [78].

The native biosynthesized polymer of gellan consists of two residues of β-D-glucose, one of β-D-glucuronate and one of α-L-rhamnose, which provides hydroxyl groups, as well as the L-glyceryl and acetyl substituents positioned at O(2) and O(6) of D-glucose residue of the tetrasaccharide sequence [79].

Bergmann et al. [80] suggested that the bivalent heavy metals ions (such as manganese(II), cadmium(II), and lead(II)) link to xanthan via forming the intramolecular complex between the side chains of the same xanthan molecule, solely by the complexing with the pyruvate units that are located at the outer ends of the side chains. The involvement of carboxylate groups of the adjacent glucuronic acid residues in the binding of the lighter bivalent cations (e.g., calcium(II)) has been proposed by Tako and Nakamura [81]. Ciesielski and Tomasik [82] demonstrated that carboxylic groups of xanthan polymer are preferably involved in the ligation of copper(II), cobalt(II), and iron(III), forming octahedral or square planar structures. In gellan, the bivalent cations form intermolecular bridges via ionic bonding between the carboxyl oxygen atoms of the D-glucuronosyl residues on different polysaccharide molecules [83,84].

The character of the interaction of the active functional groups of the polysaccharide with metallic ions also affects the properties of polymers, e.g., polymer morphology and solution rheology [85]. Some polysaccharides provide intramolecular binding sites for the cations, and their binding results in a considerable reduction of the polymer’s hydrodynamic radius, and thus decreases the viscosity of the solution. Other polysaccharides form chelate-like complexes with metal cations via intermolecular cross-linking, which leads to a vast increase in the solution viscosity [86]. The ability to create cross-linking networks enables the biopolymers to encapsulate the contaminants. The cross-linking of biopolymers also improves the mechanical properties of soils and the durability of earthen construction materials [87]. Since the polysaccharide chains bind both the metallic ions and soil particles, they can stabilize metals in subsurface soils by trapping the contaminants in stable complexes, while enhancing the soil properties. Xanthan has been shown to decrease permeability and increase the shear strength of the sand and improved the uptake ability of copper(II) [88], since it forms bridges between distant soil particles most likely via hydrogen bonding and cation bridging [89].

## 5. Immobilization of Heavy Metals by Native and Modified Xanthan and Gellan

Although both xanthan and gellan are usually used in the food industry, they also show great prospects in the remediation of contaminated waters, since they provide various active functional groups that are capable of binding the heavy metals from aqueous solutions and removing them efficiently [90]. Wang et al. [91] studied the potential applicability of low-viscosity-type xanthan gum produced by *X. campestris* CCTCC M2015714 in the detoxication of lead(II)-, cadmium(II)-, and copper(II)-contaminated waters at acidic pH simulating the stomach pH. The xanthan showed excellent adsorption capacity by reducing the concentration of contaminants in an aqueous solution by 50% after one hour. Gellan gum’s performance in nickel(II) removal from the aqueous solutions was equally excellent since its sorption capacity was found to be superior in comparison to other gel-forming polymers, including agar, Ca-alginate, silica gel, polyacrylamide, and agarose [92].

There are numerous approaches to synthesizing the gellan- and xanthan-based sorbents that are applicable for the cost-effective and environmentally friendly treatment of wastewater and which improve the thermal and mechanical performance and low surface area of the native biopolymers [93]. The heavy metal removal efficiency of native polysaccharides in the remediation of contaminated waters can be enhanced via polymer functionalization in the form of blending, grafting, or mixing with various (nano)materials to introduce an additional functional group to make modified polysaccharides more efficient for heavy metal adsorption or to enhance the mechanical properties of the adsorbent. This is usually realized via the chemical treatment of xanthan and gellan polysaccharides. In the case of xanthan gum modification, the usual applicable chemical methods include acetylation and pyruvation, etherification and esterification, acidic hydrolysis, cationic modification, grafting and cross-linking, cold-plasma treatment, and genetic modification of xanthan producers [94]; however, the cross-linking reaction and graft copolymerization have shown the most significant improvement of the heavy metal adsorption capacity and adsorbent recyclability [95]. Modification using chemical and cross-linking agents, e.g., acrylic acid, vinyl imidazole, and vinyl pyridine, generally improve the hydrogel-forming kinetics and mechanical strength of the biopolymers [96].

Modification by grafting synthetic polymers onto a native xanthan polysaccharide allows for overcoming some drawbacks of the native xanthan; also, it yields new materials with different structural characteristics and desirable properties [97]. Pandey and Mishra [98] synthesized a graft copolymer of xanthan gum and ethylacrylate, which increased the thermostability of the polymer and provided additional binding sites for zinc(II). The pure gum adsorbed 1.02 mequiv·g^−1^ of zinc(II) at pH 5.0, while the presence of polyethylacrylate grafts increased the adsorption capacity by six-fold. Later, Pandey and Mishra [99] modified the procedure for graft copolymerization to achieve higher grafting efficiency by implementing microwave irradiation. The increasing amount of binding sites on the polyethylacrylate grafts resulted in an increase of the lead(II) removal from 60 to 76.5% at pH 5.0.

The FTIR spectra revealed that the partial hydrolyzation of the product of polyacrylamide-grafted xanthan gum increased the abundance of carboxylic groups, thus suggesting the increase of anionic moieties in the polymer. This should have had a beneficial effect on the adsorbent performance in the immobilization of cationic heavy metals; however, the flocculation performance was affected negatively [100]. This restriction can be overcome by impregnating the polyacrylamide-grafted xanthan gum with nanomaterials, such as silica [101]. This polymeric nanocomposite with high hydrodynamic volume shows excellent performance in lead(II) removal from the aqueous solution. The maximum adsorption capacity of 537 mg·g^−1^ for lead(II) was calculated using the Langmuir isotherm model, highlighting the excellent adsorption efficiency of the synthesized nanocomposite for lead removal from the complex industrial wastewaters. Promising results in lead(II) removal have been achieved also by cross-linking the xanthan and guar gums, where the metal cation was predominantly attached to carboxylic groups [102]. Their availability in this hybrid polymer was regulated by the swelling of the polymer, which instigated and facilitated the lead binding inside the network sites.

Zheng et al. [103] synthesized a novel hydrogel by grafting acrylamide and partly neutralized acrylic acid onto xanthan gum with trimethylolpropane triglycidyl ether as a cross-linking agent, which has been tested for removal of copper(II) with indicated maximum sorption capacity of 130.3 mg·g^−1^. This is significantly higher than other reported copper(II) removal capacities of grafted xanthan gum, e.g., 39.1 mg·g^−1^ for the xanthan gum-*graft*-2-acrylamido-2-methyl-1-propane sulfonic acid [104] or 46.9 mg·g^−1^ for xanthan gum modified with thionyl chloride followed by ethylenediamine modification [105]. A hybrid pectin–xanthan biosorbent showed immobilization capacities comparable to a pectin-based sorbent containing gellan gum for both the lead(II) and cadmium(II) cations [106]. The sorbents’ maximum sorption capacities were approximating 0.6 and 0.7 mmol·g^−1^ for cadmium and lead cations, respectively.

Inorganic fillers in cross-linked polymers, such as clay material, can lend biopolymer-specific chemical and physical properties and minimize chain mobility. Esmaeildoost et al. [107] attempted to modify a xanthan/poly acrylic acid-based composite hydrogel with Cloisite 15A nanoclay to improve its rheological and swelling properties. Besides increasing the porosity of the novel adsorbent, it also improved polymers’ affinities towards cobalt(II), copper(II), and nickel(II) in neutral aqueous solution with sorption capacities of 436.6, 530.1, and 511.7 mg·g^−1^, respectively. Mama et al. [108] prepared xanthan-gum-based geopolymer using kaolin and palm oil fuel ash that was tested for removal of heavy metals from aqueous media. The listed sorption capacities included those of inorganic copper (35.0 mg·g^−1^), iron (45.2 mg·g^−1^), and zinc (44.6 mg·g^−1^) cations; its good performance in the ternary sorption system highlighted the applicability of this novel composite for actual wastewater treatment.

Another novel approach is the modification of biopolymers with ferromagnetic (nano)materials that allows the magnetic separation of contaminants bound to the adsorbent. This includes a Fe_3_O_4_@silica–xanthan gum composite that was successfully tested for lead(II) removal and showed promising results for resolving the critical pollution of lead-bearing battery industry wastewater [109]. Mirza and Ahmad [110] also demonstrated that the bionanocomposite of xanthan gum and montmorillonite (a clay mineral) can be successfully utilized for lead(II) removal from electroplating and battery manufacturing wastewater, achieving 80% lead-removing efficiency.

Magnetic nanoparticles are also advantageous in the remediation of heavy metals, since they can be tailored via surface functionalization for specific contaminants with organic compounds [111]. Various biopolymers have been used as microcapsules for the nanomagnetic particles’ immobilization as a natural cross-linked matrix, which also provides new negatively charged carboxyl functional groups that enhance the effectiveness of the removal of the cationic metals from wastewaters [112]. Wang et al. [113] demonstrated that the carboxylic groups of gellan in super-paramagnetic gel beads are critical for binding the lead(II), manganese(II), and chromium(III) cations from aqueous solutions. The main driving mechanism for their immobilization is ion exchange, either with calcium(II) or hydrogen cations from the cross-linked polymer. Yamada and Kametani [114] also demonstrated that the carboxyl functional groups in gellan gum (coupled to silane by 3-glycidoxypropyltrimethoxysilane) are essential for the removal of both the heavy metals and rare earth elements, while the light metal cations (e.g., magnesium(II) and aluminum(III)) are primarily bound to hydroxyl groups.

The cross-linked gellan was also used for the encapsulation of other biopolymers, such as alginate. Nussinovitch and Dagan [115] prepared a unique sorbent comprising a liquid alginate core encapsulated in a spherical polymer membrane of cross-linked gellan gum which showed a high affinity towards lead(II), with a sorption capacity of 267 mg·g^−1^, while exhibiting reasonable regenerative capacity.

Liang et al. [116] prepared a microsphere of Ca-gellan gum cross-linked with glutaraldehyde that was subsequently modified by ethanol to enhance the porosity of the adsorbent. This proved to be beneficial for the removal of uranyl cations (UO_2_^2+^), and the maximum sorption capacity of the novel biosorbent was 202.8 mg·g^−1^. The same research team also synthesized a gellan-gum-based adsorbent with less, a 97.9 mg·g^−1^ removal capacity for uranyl cations, but with improved gel strength, by reinforcing gellan gum microspheres using the carboxymethyl konjac glucomannan [117].

The research outcomes indicate that although natural xanthan and gellan provide plenty of carboxyl and hydroxyl groups for heavy metal immobilization, their adsorption capacity, selectivity, and mechanical properties (e.g., swelling and metal-induced gelling ability, mechanical and thermal stability) can be profoundly improved by the grafting of other functional groups or molecules onto the biopolysaccharide structure. Therefore, even in cases when xanthan- and gellan-derived adsorbents possess lower sorption capacities in comparison to other conventional or non-conventional adsorbents (Table 1), there is always an option for the proper chemical functionalization of xanthan and gellan to develop a new green adsorbent that will be more suitable for specific heavy metal or wastewater treatment conditions. Thus, there are many reports targeting the improvement of the sorptive and mechanical properties of xanthan and gellan via chemical or physical means. However, the attractiveness of the natural polymers in remediation of heavy-metal-contaminated waters also includes their low cost (and availability) [118], and since the economic viability of the final adsorbent fabrication is governed by the number of steps in its synthesis [119], any chemical post-modification of xanthan and gellan increases the production costs and, thus, diminishes the cost-effectiveness of the whole process.

To summarize the presented outcomes, the practical application of xanthan and gellan polymers in heavy metal removal from aqueous media may be successful, yet uncertain. This is due to high competitiveness among available non-conventional adsorbents regarding their sorptive performance and costs. This is clearly exemplified in Table 1, which depicts various economically feasible and eco-friendly alternatives to xanthan and gellan, which are performing equally or even better in the removal of heavy metals from aqueous media (Table 1).

## 6. Other Implications of Xanthan and Gellan in Remediation of Heavy-Metal-Contaminated Sites

Besides immobilization via sorptive interactions, the application of xanthan and gellan gums in heavy metal remediation is limited, primarily due to the non-degradative properties of heavy metals. There is a much wider variety of strategies for the decontamination of degradable organic pollutants using polymeric bioinspired materials [154]. Still, heavy metals can undergo conversion into new species whose toxicity and bioavailability may differ depending on the nature of their transformation. McCarthy et al. [155] used xanthan gum to encapsulate the whole cells of *Pseudomonas veronii* and attached the biopolymer mix onto zeolite granules. Bioaugmentation of mercury-contaminated soil with immobilized bacteria having mercury(II) reduction and volatilization capacity resulted in a sharp increase of gaseous elemental mercury emissions, with maximum flux rates exceeding 10 μg Hg·m^2^·h^−1^.

Xanthan and gellan are both viscous biopolymers; hence, as an additive, they can enhance soil strength. Chang et al. [156] demonstrated that the interaction of xanthan polymer with soil particles enhances the compressive strength of soil by increasing the cohesive forces within the soil. More specifically, xanthan acts as a cementitious binder that forms firm xanthan–soil matrices. This also affects the extractability of the heavy metals from contaminated soils that are amended with biopolymers, e.g., the xanthan addition into the soil significantly decreases the copper, lead, and zinc extractability (using HCl and NaH_2_PO_4_) [157].

A unique approach combining plant growth and amendment of xanthan gum has been suggested for remediation purposes of heavy-metal-contaminated soils by Ni et al. [158], who noted a significant (over 50%) decrease in soil heavy metal concentrations when a xanthan soil content of 0.25% was applied on clay soil with growing oat. Apart from enhancing the soil strength, the biopolymer–plant–soil reinforcing method was also able to improve the soil environment.

## 7. Concluding Remarks

Biopolymer-based materials are rapidly emerging as an effective green alternative to traditional adsorbents employed for heavy metal removal. As our review shows, among the other commercially available biopolymers, the heteropolysaccharides xanthan and gellan show great prospects in this regard. Unfortunately, the actual research on their performance in heavy metal removal is still in the initial stages and comprises developing new, more effective xanthan- or gellan-based adsorbents and their testing under optimized laboratory conditions. Nonetheless, to evaluate the viability and cost-effectiveness of xanthan and gellan’s employment in remediation technologies, experiments should be performed at a larger scale and should involve more complex matrices (e.g., mine drainage). However, no such studies are available to this day. Still, we hope it will not be left unchallenged and in time to come, this issue will be overcome by reporting an in situ large-scale experiment that would highlight the economic feasibility and excellent performance of xanthan- and gellan-based adsorbents for the removal of heavy metals from contaminated waters.

## Figures and Tables

**Table 1:** Reported maximum sorption capacities of xanthan- and gellan-based sorbents along with other conventional and non-conventional sorbents for lead(II) (the values indicate the maximum sorption capacities calculated from Langmuir isotherm).

Sorbent	Sorption Capacity (mmol·g^−1^)	Reference
***Xanthan- or gellan-based sorbents***		
Fe_3_O_4_@silica–**xanthan gum** composite	0.10	Peng et al. [109]
photocrosslinked **xanthan gum** and guar gum	0.47	Pal et al. [102]
microwave-synthesized **xanthan gum**-g-poly(ethylacrylate)	0.69	Pandey and Mishra [99]
hybrid pectin-based biosorbents containing **xanthan** (or **gellan**)	0.73 (0.78)	Jakóbik-Kolon et al. [106]
**gellan gum** gel beads	0.85	Lázaro et al. [92]
**xanthan gum**/montmorillonite bionanocomposite	0.90	Mirza and Ahmad [110]
magnetic **gellan gum** gel beads	1.26	Wang et al. [113]
nanosilic-filled **xanthan gum** grafted with polyacrylamide	2.59	Ghorai et al. [101]
***Microbial- and algal-biomass-derived sorbents***		
bacterial strain *Stenotrophomonas maltophilia* with phosphorus-accumulating effects	0.42	Li et al. [120]
*Bacillus* sp. isolated from activated sludge	0.54	García et al. [121]
*Staphylococcus aureus* treated with magnetic Fe_3_O_4_-phthalate nanoparticles	0.95	Mahmoud et al. [122]
brown algae *Ascophyllum nodosum* and *Fucus spiralis*	0.98	Romera et al. [123]
baker yeast biomass grafted with polyamic acid	0.99	Yu et al. [124]
SiO_2_-nanoparticle-immobilized *Penicillium funiculosum*	1.27	Mahmoud et al. [125]
filamentous fungus *Aspergillus piperis*	1.33	de Wet and Brink [126]
fungus *Penicillium purpurogenum*	1.38	Say et al. [127]
marine alga *Ulva* sp.	1.46	Sheng et al. [128]
marine macroalga *Durvillaea potatorum*	1.55	Yu et al. [129]
sponge-like biosorbent of *Pseudomonas putida*	1.67	Wang et al. [130]
***Biopolymer-based adsorbents***		
carboxylated-cellulose-nanofibril-filled magnetic chitosan hydrogel beads	0.85	Zhou et al. [131]
alginate extracted from marine alga *Sargassum filipendula*	1.16	Kleinübing et al. [132]
extracellular polymeric substances from *Leuconostoc citreum* immobilized in Ca-alginate	1.30	Xu et al. [133]
chitosan/silica gel composite	2.22	Rajiv Gandhi and Meenakshi [134]
extracellular polymeric substances isolated from activated sludge (protein 48.7%, polysaccharide 31.3%, and nucleic acid 20.0%)	2.84	Zhou et al. [135]
extracellular polymeric substances from *Agrobacterium tumefaciens*	3.44	Cui et al. [136]
poly(acrylic acid) grafted and glutaraldehyde-crosslinked chitosan	3.54	Ge et al. [137]
***Agricultural waste derived sorbents***		
banana peels	0.01	Anwar et al. [138]
chitosan-pyromellitic-dianhydride-modified biochar	0.07	Deng et al. [139]
formaldehyde-treated orange peels	0.20	Lugo-Lugo et al. [140]
magnetic peanut husk functionalized with iminodiacetic acid	0.34	Aryee et al. [141]
Chinese medicine material residue biochars	0.40	Wang et al. [142]
magnetic thioamide-functionalized biochar from date leaves and stalks	0.50	Zahedifar et al. [143]
carbon prepared from peanut husks	0.87	Ricordel et al. [144]
magnetic wheat straw biochar	1.44	Gong and Chi [145]
***Conventional adsorbents***		
magnetite nanoparticles	0.11	Wang et al. [146]
commercial activated carbon	0.23	Krishnan et al. [147]
Turkish illitic clay	0.26	Ozdes et al. [148]
activated alumina	0.40	Naiya et al. [149]
natural zeolitic tuff	0.43	Perić et al. [150]
zeolite	0.66	Pfeifer et al. [151]
mixture of zeolite, bentonite, and kaolin	0.68	Salem and Akbari Sene [152]
HCl-activated Aloji clay	1.61	Obayomi and Auta [153]

## Data Availability

Not applicable.
